# Tumor-Targeting *Salmonella typhimurium* A1-R Arrests a Chemo-Resistant Patient Soft-Tissue Sarcoma in Nude Mice

**DOI:** 10.1371/journal.pone.0134324

**Published:** 2015-08-03

**Authors:** Yukihiko Hiroshima, Ming Zhao, Yong Zhang, Nan Zhang, Ali Maawy, Takashi Murakami, Sumiyuki Mii, Fuminari Uehara, Mako Yamamoto, Shinji Miwa, Shuya Yano, Masashi Momiyama, Ryutaro Mori, Ryusei Matsuyama, Takashi Chishima, Kuniya Tanaka, Yasushi Ichikawa, Michael Bouvet, Itaru Endo, Robert M. Hoffman

**Affiliations:** 1 AntiCancer, Inc., San Diego, California, United States of America; 2 Department of Surgery, University of California San Diego, San Diego, California, United States of America; 3 Department of Gastroenterological Surgery, Yokohama City University Graduate School of Medicine, Yokohama, Japan; University of Nebraska Medical Center, UNITED STATES

## Abstract

A patient-derived nude-mouse model of soft-tissue sarcoma has been established and treated in the following groups: (1) untreated controls; (2) gemcitabine (GEM) (80 mg/kg, ip, weekly, 3 weeks); (3) Pazopanib (100 mg/kg, orally, daily, 3 weeks) and (4) *Salmonella typhimurium* A1-R (5 × 10^7^ CFU/body, ip, weekly, 3 weeks). The sarcoma was resistant to GEM (p = 0.879). Pazopanib tended to reduce the tumor volume compared to the untreated mice, but there was no significant difference (p = 0.115). *S*. *typhimurium* A1-R significantly inhibited tumor growth compared to the untreated mice (p = 0.001). *S*. *typhimurium* A1-R was the only effective treatment for the soft-tissue sarcoma nude mouse model among all treatments including a newly approved multiple tyrosine kinase inhibitor; Pazopanib. These results suggest tumor-targeting *S*. *typhimurium* A1-R is a promising treatment for chemo-resistant soft-tissue sarcoma.

## Introduction

Soft-tissue sarcomas are rare mesenchymal cancers comprising approximately 50 histological types [1]. The yearly incidence of soft-tissue sarcomas in the USA is 11,280 cases, with 3,900 deaths. Patients with metastatic soft-tissue sarcomas have a median overall survival of about 12 months.

Pazopanib (Votrient) is a multiple tyrosine kinase inhibitor approved for the treatment of advanced (unresectable and/or metastatic) soft-tissue sarcomas in patients who have received prior chemotherapy. Pazopanib improved progression-free survival in a Phase III clinical trial [2].

An attenuated strain of Clostridium novyi (C. novyi-NT) was used to treat a human patient who had an advanced leiomyosarcoma. Treatment was intratumor (i.t.) injection of C. novyi-NT spores. C. novyi-NT regressed the tumor within and surrounding the bone [3].

Our laboratory has previously developed a genetically-modified bacteria strain, *Salmonella typhimurium* A1-R, selected for tumor-targeting in vivo. *S*. *typhimurium* A1-R is auxotrophic for leu and arg [4]. The strain targets and grows in tumors. In contrast, normal tissue is cleared of these bacteria even in immunodeficient athymic mice. *S*. *typhimurium* A1-R is effective against prostate cancer [5], breast cancer [6, 7], pancreatic cancer [8–11], glioma [12, 13], lung cancer [14], fibrosarcoma [15] and osteosarcoma [16].

In the present study, we report the efficacy of *S*. *typhimurium* A1-R against a human patient chemo-resistant sarcoma growing in nude mice. Tumors growing in nude mice can faithfully replicate important features of the patient’s cancer, such as tumor markers even after passage [17, 18].

## Materials and Methods

### Animals

Male athymic *nu/nu* nude mice (AntiCancer Inc., San Diego, CA), 4–6 weeks old, were used in this study. Mice were kept in a barrier facility under HEPA filtration. Mice were fed with autoclaved laboratory rodent diet. All mouse surgical procedures and imaging were performed with the animals anesthetized by intramuscular injection of 50% ketamine, 38% xylazine, and 12% acepromazine maleate (0.02 ml solution). All animal studies were conducted with an AntiCancer Institutional Animal Care and Use Committee (IACUC)-protocol specifically approved for this study and in accordance with the principals and procedures outlined in the National Institute of Health Guide for the Care and Use of Animals under Assurance Number A3873-1.

### Specimen collection

The patient provided informed written consent and samples were procured and the study was conducted under the approval of the Institutional Review Board of the UC San Diego Medical Center.

### Establishment of patient nude mouse model of sarcoma

Tumor tissues were obtained from the patient with a metastatic soft-tissue sarcoma of the retroperitoneum at biopsy and cut into fragments (3-mm^3^) and transplanted subcutaneously in nude mice. Tumors in the present study were in their second passage.

### Preparation of bacteria

*S*. *typhimurium* A1-R was grown overnight on LB medium and then diluted 1:10 in LB medium. Bacteria were harvested at late-log phase, washed with PBS, and then diluted in PBS. Bacteria were then used for experiments [6].

### Treatment of soft-tissue sarcoma in nude mice

The patient sarcoma established in nude mice was passaged subcutaneously to 20 nude mice to determine the efficacy of various treatments. Four weeks after implantation, the mice in each model were randomized to 6 mice in the untreated control and 4 groups of 5 mice in each treatment group and treated as follows: (1) untreated control; (2) gemcitabine (GEM, Eli Lilly and Company, Indianapolis, IN, USA) (80 mg/kg, ip, weekly, 3 weeks); (3) Pazopanib (Selleck Chemicals, Houston, TX, USA, 100 mg/kg, orally, daily, 3 weeks) and (4) *S*. *typhimurium* A1-R (5 × 10^7^ CFU/body, ip, weekly, 3 weeks). Tumor size was evaluated every 3 to 4 days by caliper measurements. The approximate volume of the mass was calculated using the formula 4/3π• (d/2)^2^• D/2, where d is the minor tumor axis and D is the major tumor axis. Body weight of the mice was measured on a balance once a week. Relative tumor volume and body weight were calculated by comparison to Day 1. The endpoint of the experiment was when tumor size in the untreated control mice became approximately 2 cm. The method of euthanasia was CO_2_ inhalation.

### Tissue histology

Tumor samples were removed with surrounding normal tissues at the time of resection. Fresh tissue samples were fixed in 10% formalin and embedded in paraffin before sectioning and staining. Tissue sections (5 μm) were deparaffinized in xylene and rehydrated in an ethanol series. Hematoxylin and eosin (H & E) staining was performed according to standard protocols. The sections were examined using a BH-2 microscope (Olympus, Tokyo, Japan) equipped with a INFINITY1 2.0 megapixel CMOS digital camera (Lumenera Corporation, Ottawa, Canada). All images were acquired using INFINITY ANALYZE software (Lumenera Corporation) without post-acquisition processing.

### Evaluation of histopathological response to treatment

Histopathological response to treatment was defined according to Evans’s grading scheme: Grade I, little (<10%) or no cancer cell destruction is evident; Grade IIa, destruction of 10%-50% of cancer cells; Grade IIb, destruction of 51%-90% of cancer cells; Grade III, few (<10%) viable-appearing cancer cells are present; Grade IV, no viable cancer cells are present [11, 19].

### Confocal imaging of *S*. *typhimurium* A1-R-GFP in sarcoma tissue

Resected sarcoma specimens from mice treated with *S*. *typhimurium* A1-R were embedded with optimal cutting temperature (OCT) compound (Tissue-Tek; Sakura Finetek Europe BV, Zoeterwude, Netherlands) and preserved in liquid nitrogen. Frozen sections of 7–10 μm thickness were prepared with a CM1850 cryostat (Leica, Wetzlar, Germany). The frozen sections were directly observed with a confocal microscope (Fluoview FV1000, Olympus, Tokyo, Japan). Excitation sources were semiconductor lasers at 473 nm for GFP excitation. After confocal imaging, frozen sections were fixed in 10% formalin and H & E staining was performed.

### Culture of GFP-labeled *S*. *typhimurium* A1-R bacteria from tumors and organs

Subcutaneous tumors and normal nude mouse organs (blood and liver) were removed at the termination of the treatment experiments. Bacteria were isolated from the tumors and organs and cultured in LB agar for 24 hours, and imaged with the OV100 small animal imaging system (Olympus, Tokyo, Japan) [20].

### Statistical analysis

PASW Statistics 18.0 (SPSS, Inc) was used for all statistical analyses. The Student’s t-test was used to compare continuous variables between two groups. A p value of 0.05 was considered statistically significant for all comparisons.

## Results and Discussion

### Soft-tissue patient sarcoma grown in nude mice recapitulates the histology of the original tumor

**Fig 1:**
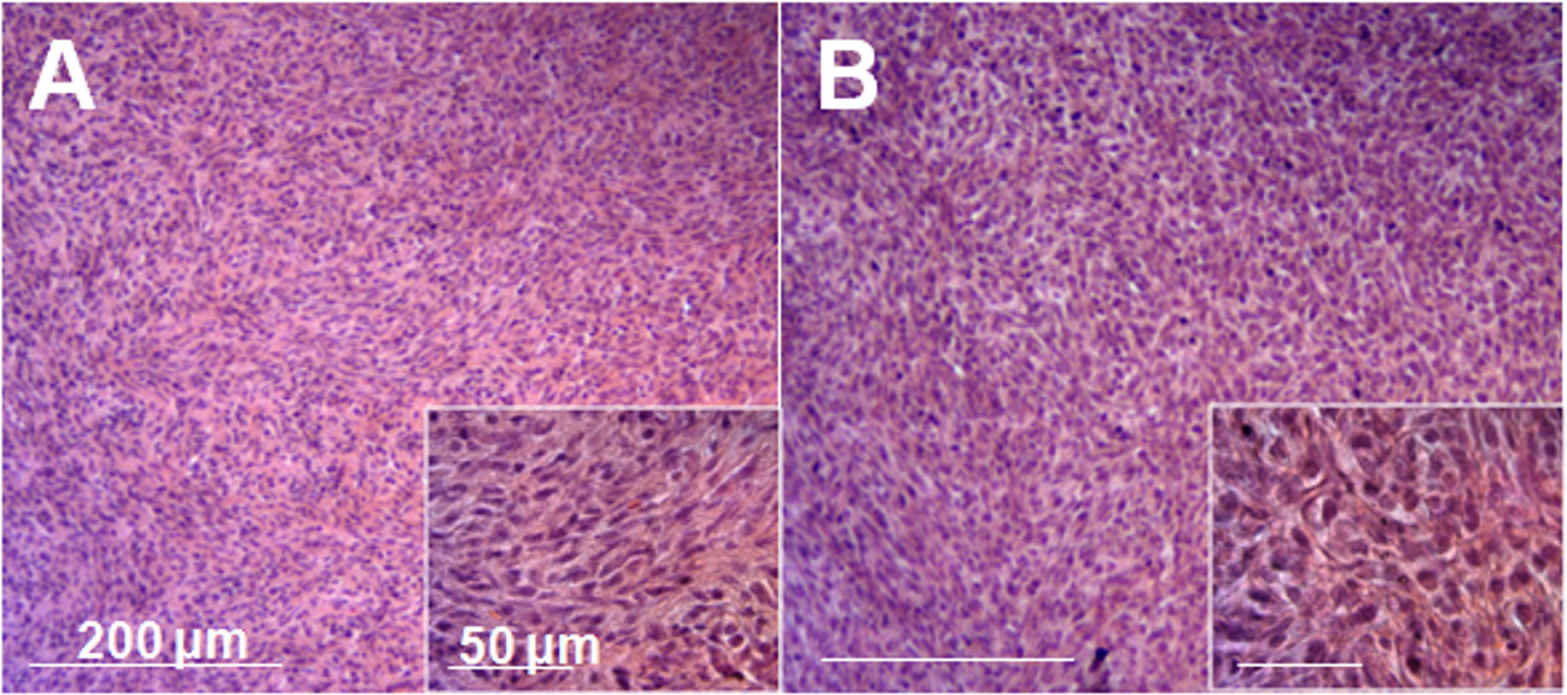
Tumor histology. **A)** Histology of the original patient sarcoma. **B)** Histology of the mouse grown sarcoma.

The majority of the original-tumor section was comprised of sarcomatous high grade spindle cells of varying sizes, demonstrating abundant, finely granular cytoplasm and atypical, pleomorphic, round–to-elongated nuclei with irregular nuclear membranes, an open chromatin pattern and prominent nucleoli (Fig 1A). The mouse-grown tumors had histological structures similar to the original tumor (Fig 1B).

### *S*. *typhimurium* A1-R is highly effective on the patient soft-tissue sarcoma in nude mice

**Fig 2:**
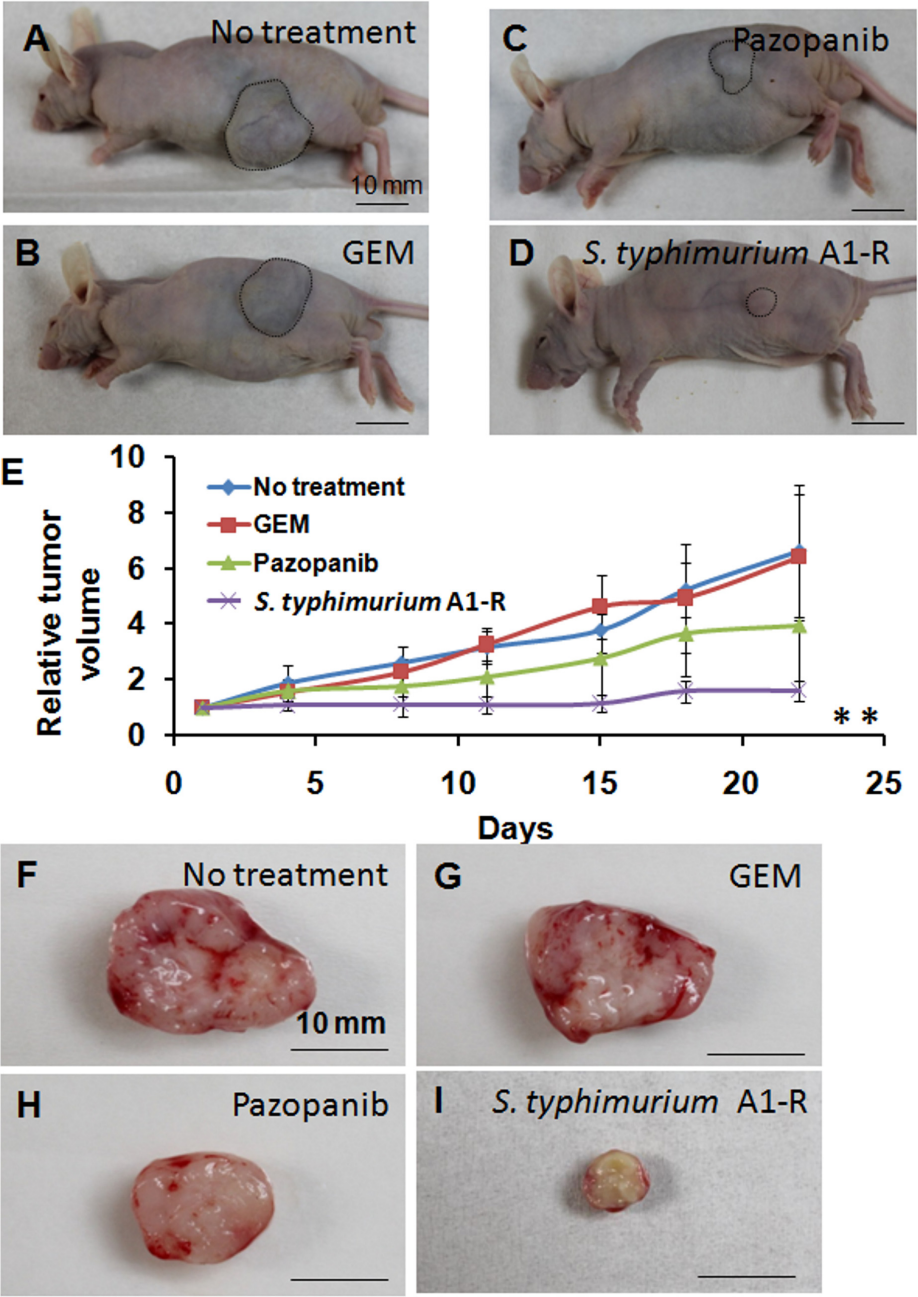
Drug-response of soft tissue sarcoma in nude mice. A representative image of nude mouse with the subcutaneous sarcoma in **(A)** the untreated mice; **(B)** GEM-treated; **(C)** Pazopanib-treated or **(D)** *Salmonella typhimurium* A1-R-treated. Scale bars: 10 mm. **(E)** Growth curves of the subcutaneous sarcoma’s treated with various drugs as described above. The values are mean relative tumor volume ± S.D. (bars) of five different tumors. ** p < 0.01, compared to no treatment group. **(F-G)** Representative cross-sections of excised subcutaneous tumors from the control and treatment groups with type of treatment indicated.

The relative tumor volume on day 22, compared to day 1, of each group was as follows: (1) untreated control: 6.61 ± 2.37; (2) GEM: 6.39 ± 2.26; (3) Pazopanib: 3.94 ± 2.71; (4) *S*. *typhimurium* A1-R: 1.58 ± 0.37 (Fig 2). The sarcoma did not significantly respond to GEM (p = 0.879). Pazopanib tended to reduce the tumor volume compared to the untreated mice, but there was not a significant difference (p = 0.115). *S*. *typhimurium* A1-R significantly inhibited the sarcoma tumor growth compared to the untreated control mice (Fig 2) (p = 0.001). Sizes of all tumors are listed in the S1 Table. No body weight loss was found in any treatment groups.

### Histological response to treatment

**Fig 3:**
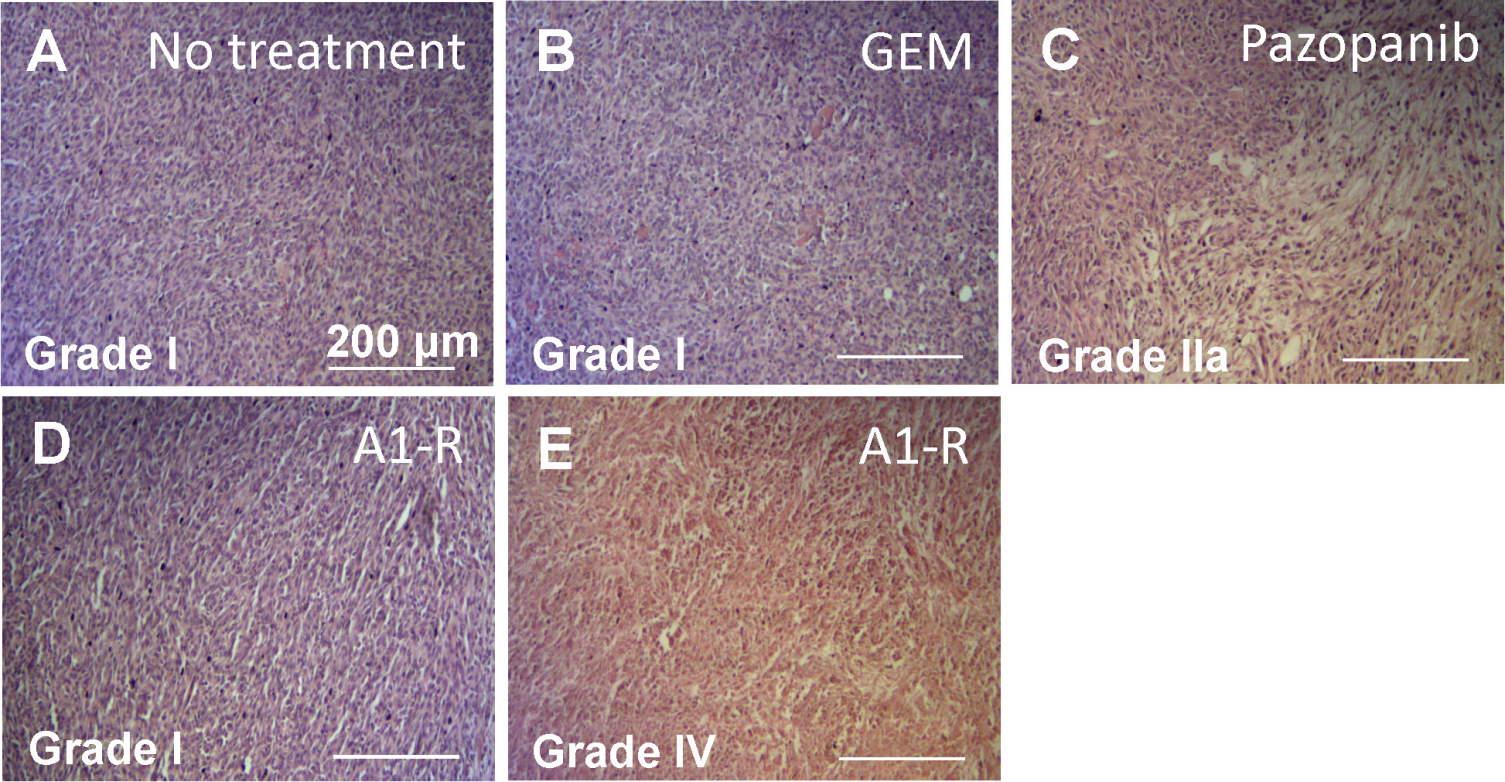
Effect of treatment on histology in nude mice. Histopathological responses to treatments were defined according to Evans’s grading scheme. **(A)** Treatment effect of control / no treatment was judged as grade I; **(B)** GEM treatment as grade I; **(C)** Pazopanib treatment as grade IIa; **(D)** *S*. *typhimurium* A1-R of a viable-like area as grade I and **(E)** *S*. *typhimurium* A1-R of a necrotic area as grade IV. Scale bars: 200 μm.

Histopathological response to each treatment was defined according to Evans’s grading scheme. In the control (no treatment) and GEM-treated sections, the tissue sections from the tumor were occupied by viable cancer cells (Fig 3A and 3B). Approximately 40% of cancer cells were destroyed and replaced by stromal cells in the tumor sections treated with Pazopanib (Fig 3E). Tumors treated with *S*. *typhimurium* A1-R consisted of 2 components; one was a viable-like component and the other one was a necrotic component. The viable-like component was occupied by cancer cells (Fig 3D). In contrast, no viable cancer cell were detected in a necrotic component of the *S*. *typhimurium* A1-R-treated tumor (Fig 3E). Although the viable cancer cells were found, they did not form a tumor as can be seen from Fig 2. The untreated control and GEM were judged as grade I; Pazopanib as IIa; *S*. *typhimurium* A1-R of a viable-like component as grade I and the necrotic component as IV. The necrotic component was not detected in any treatment group except for *S*. *typhimurium* A1-R (Fig 3A–3E). Tumor heterogeneity may be a factor in the observed chemoresistance of the soft tissue sarcoma that was overcome by *S*. *typhimurium* A1-R.

**Fig 4:**
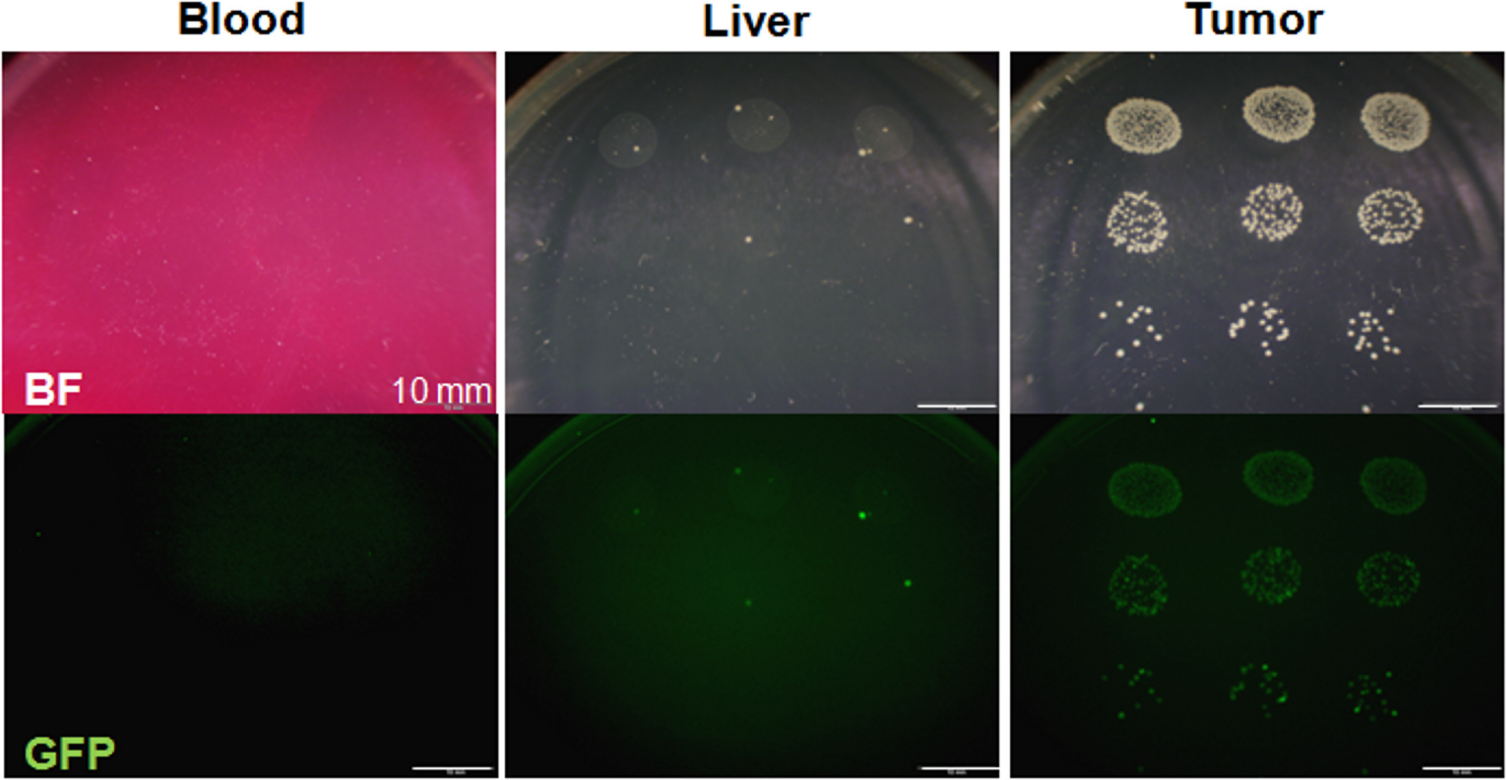
Tumor-targeting of *S*. *typhimurium* A1-R. Distribution of GFP-labeled *S*. *typhimurium* A1-R in tumors and organs. Representative images of GFP-labeled *S*. *typhimurium* A1-R bacteria culture isolated from the tumor and the normal organs (blood and liver) of the mice treated with *S*. *typhimurium* A1-R. GFP-labeled *S*. *typhimurium* A1-R were clearly detected only in the tumor. Only a few GFP-labeled *S*. *typhimurium* A1-R were detected in the liver and no GFP-labeled *S*. *typhimurium* A1-R was detected in blood. Scale bars: 10 mm.

GFP-labeled *S*. *typhimurium* A1-R was isolated from the tumor (Fig 4), but not from blood and only minimally from liver, indicating the tumor was effectively targeted by *S*. *typhimurium* A1-R.

*S*. *typhimurium* A1-R was the only effective treatment for the soft-tissue patient sarcoma growing in nude mice including GEM and a newly approved multiple tyrosine kinase inhibitor; Pazopanib. One factor in chemoresistance of solid tumors is that the majority of the cancer cells within the tumor are in a chemoresistant G_0_/G_1_ quiescent cell-cycle phase [21]. We have recently shown that quiescent cancer cells are sensitive to *S*. *typhimurium* A1-R [22]. *S*. *typhimurium* A1-R, unlike C. novyi-NT, is a facultative anaerobe and can be administered systemically such as in the present study (i.p.), whereas C. novyi-NT seems to require i.t. administration which makes it different to target metastasis. The results of the present study indicate tumor-targeting *S*. *typhimurium* A1-R is a promising treatment for soft-tissue sarcomas. In future experiments, *S*. *typhimurium* A1-R will be tested in a series of patient soft tissue sarcoma grown in mice as a bridge to the clinic, where we also intend to focus on sarcoma.

Evaluation of biomarker expansion under various treatment regimens will be evaluated in future experiments.

## Supporting Information

S1 Table Tumor sizes of all mice on Day-22. (DOCX)
