# Supplementary material for: Tumor-Targeting Salmonella typhimurium A1-R Arrests a Chemo-Resistant Patient Soft-Tissue Sarcoma in Nude Mice
Source: PLoS One. 2015 Aug 3;10(8):e0134324. doi: 10.1371/journal.pone.0134324 (PMC4523197; doi:10.1371/journal.pone.0134324)
Supplement: S1 Table — (DOCX) [file pone.0134324.s001.docx]

**Supporting Information S1 Table.** Tumor sizes of all mice on Day-22

| **No treatment** | | **GEM** | | **Pazopanib** | | ***S. typhimurium* A1-R** | |
| --- | --- | --- | --- | --- | --- | --- | --- |
| D (mm) | d (mm) | D (mm) | d (mm) | D (mm) | d (mm) | D (mm) | d (mm) |
| 20.8 | 16.2 | 21.9 | 20.7 | 18.3 | 17.5 | 13.0 | 12.8 |
| 18.6 | 17.0 | 15.8 | 13.4 | 14.2 | 13.0 | 12.2 | 9.2 |
| 17.4 | 12.8 | 14.6 | 13.5 | 12.5 | 11.1 | 8.8 | 7.6 |
| 20.8 | 19.1 | 14.9 | 14.1 | 10.2 | 9.3 | 9.1 | 8.6 |
| 14.3 | 13.0 | 15.7 | 14.7 | 11.8 | 10.5 | 9.6 | 9.1 |
| 15.5 | 14.4 |  |  |  |  |  |  |

D = longer diameter; d = shorter diameter.
